# Protective Effect of Water-Soluble Acacetin Prodrug on APAP-Induced Acute Liver Injury Is Associated with Upregulation of PPARγ and Alleviation of ER Stress

**DOI:** 10.3390/ijms241411320

**Published:** 2023-07-11

**Authors:** Jiaen Miao, Shujun Yao, Hao Sun, Zhe Jiang, Zhe Gao, Jia Xu, Kuihao Chen

**Affiliations:** 1 Department of Pharmacology, Ningbo University School of Medicine, 818 Fenghua Rd., Ningbo 315100, China; 2 The Department of Pharmacy, The First Affiliated Hospital of Ningbo University, Ningbo 315000, China; gaozhe@nbu.edu.cn

**Keywords:** acacetin, APAP, ER stress, PPARγ, liver injury

## Abstract

A water-soluble acacetin prodrug has been synthesized and reported by our group previously. Acetaminophen (APAP) overdose is a leading cause of acute liver injury. We found that subcutaneous injection of acacetin prodrug (5, 10, 20 mg/kg) decreased serum ALT, AST, and ALP, corrected the abnormal MDA and GSH in liver, and improved intrahepatic hemorrhage and destruction of liver structures in APAP (300 mg/kg)-treated mice. Molecular mechanism analysis revealed that the expressions of endoplasmic reticulum (ER) stress markers ATF6, CHOP, and p-PERK, apoptosis-related protein BAX, and cleaved caspase 3 were decreased by acacetin in a dose-dependent manner in vivo and in vitro. Moreover, via the acacetin-upregulated peroxisome-proliferator-activated receptor gamma (PPARγ) of HepG2 cells and liver, the suppressive effect of acacetin on ER stress and apoptosis was abolished by PPARγ inhibitor (GW9662) or PPARγ-siRNA. Molecular docking revealed that acacetin can bind to three active pockets of PPARγ, mainly by hydrogen bond. Our results provide novel evidence that acacetin prodrug exhibits significant protective effect against APAP-induced liver injury by targeting PPARγ, thereby suppressing ER stress and hepatocyte apoptosis. Acacetin prodrug is likely a promising new drug candidate for treating patients with acute liver injury induced by APAP.

## 1. Introduction

APAP overdose is the major cause of acute liver injury and death in the United States and Western countries [1,2,3,4]. In the United States alone, APAP overdose accounts for 46% of all acute liver failure cases [5,6]. In vivo, about 5–9% of APAP is metabolized by cytochrome P450 enzymes 2E1 (CYP 2E1) into the highly reactive toxic metabolite N-acetyl-para-benzo-quinone imine (NAPQI), which is normally detoxified by binding with glutathione (GSH) [7,8]. However, excessive NAPQI accumulates because of depletion of GSH following an APAP overdose. It is well known that excessive NAPQI binding to mitochondrial proteins, subsequently leading to mitochondrial oxidative stress and dysfunction, is the predominant mechanism of APAP-induced hepatotoxicity [9,10]. Other cellular processes also have been proven to be involved in the pathogenesis of AILI, including endoplasmic reticulum (ER) stress, activation of the NLRP3 inflammasome, and microcirculatory dysfunction [11,12,13,14]. N-acetyl cysteine (NAC) is the only approved medication for treatment of APAP overdose. The protective effect of NAC against AILI depends on its elimination of NAPQI as much as possible [15,16]. However, NAC also has some disadvantages, including adverse effects, narrow therapeutic window, and poor efficacy on patients poisoned more than 8 h [17,18]. As such, new effective drugs with fewer side effects are required to improve clinical outcomes. Since the important role of ER stress in AILI has been documented, suppression of ER stress may be a promising therapeutic strategy for treating AILI [11,19,20,21].

Acacetin is a di-hydroxy and mono-methoxy flavone reported to be present in 92 plants. The biological activities of acacetin in anti-cancer, anti-obesity, anti-diabetic, anti-inflammatory, anti-oxidant, and anti-microbial roles were well reviewed recently [22]. Considering the poor water solubility of acacetin, our previous group synthesized a water-soluble phosphate prodrug which could be converted into acacetin in vivo and prevent the induction of experimental atrial fibrillation in dogs and exhibit cardioprotective efficacy against ischemia/reperfusion injury in rats [23,24]. It has been reported that acacetin protects against D-galactosamine (D-GaIN)/LPS-induced liver injury by suppressing TLR4 signaling and enhancing autophagic flux [25]. Pretreatment of another natural flavone compound apigenin could prevent the D-GaIN/LPS-induced liver injury. The mechanism might be associated with upregulation of peroxisome-proliferator-activated receptor-γ (PPARγ) and nuclear factor erythroid 2-related factor 2 (Nrf-2) [26]. However, whether acacetin prodrug has the same effect as acacetin on AILI and the underlying mechanism remains unclear.

It was found that acacetin prodrug administrated by subcutaneous injection effectively lowered the elevation of serum ALT, AST, and ALP and restored the decrease of liver GSH in APAP-treated mice, and protected APAP-induced liver damage. Mechanistically, APAP-induced ER stress and downregulation of PPARγ was improved in acacetin-prodrug-treated mice. More importantly, PPARγ was proven to be essential for suppression of ER stress by acacetin because the protective effect of acacetin was abolished by PPARγ siRNA or the specific inhibitor. Meanwhile, computer molecular docking uncovered the interaction between acacetin and PPARγ, supporting the hypothesis that PPARγ is the target of acacetin. Our present study indicates the potential application of acacetin prodrug against AILI in clinic in future.

## 2. Results

### 2.1. Acacetin Protects APAP-Treated HepG2 Cells

**Figure 1:**
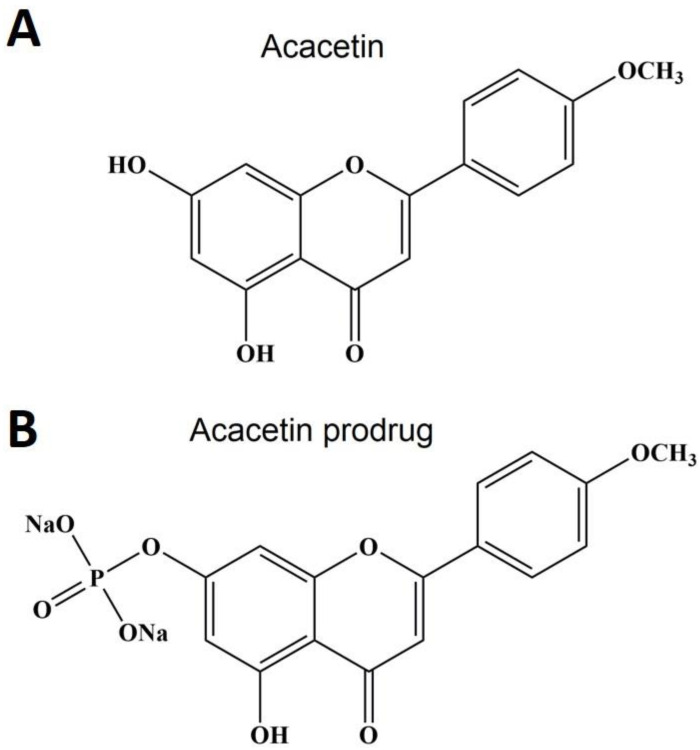
Structures of acacetin and acacetin prodrug. (**A**) The molecular formula and weight of acacetin are C_16_H_12_O_5_ and 284.26 g/mol, respectively. (**B**) Acacetin prodrug is the phosphate sodium salt of acacetin. The molecular formula and weight of acacetin prodrug are C_16_H_11_O_8_PNa_2_ and 408.21 g/mol, respectively.

**Figure 2:**
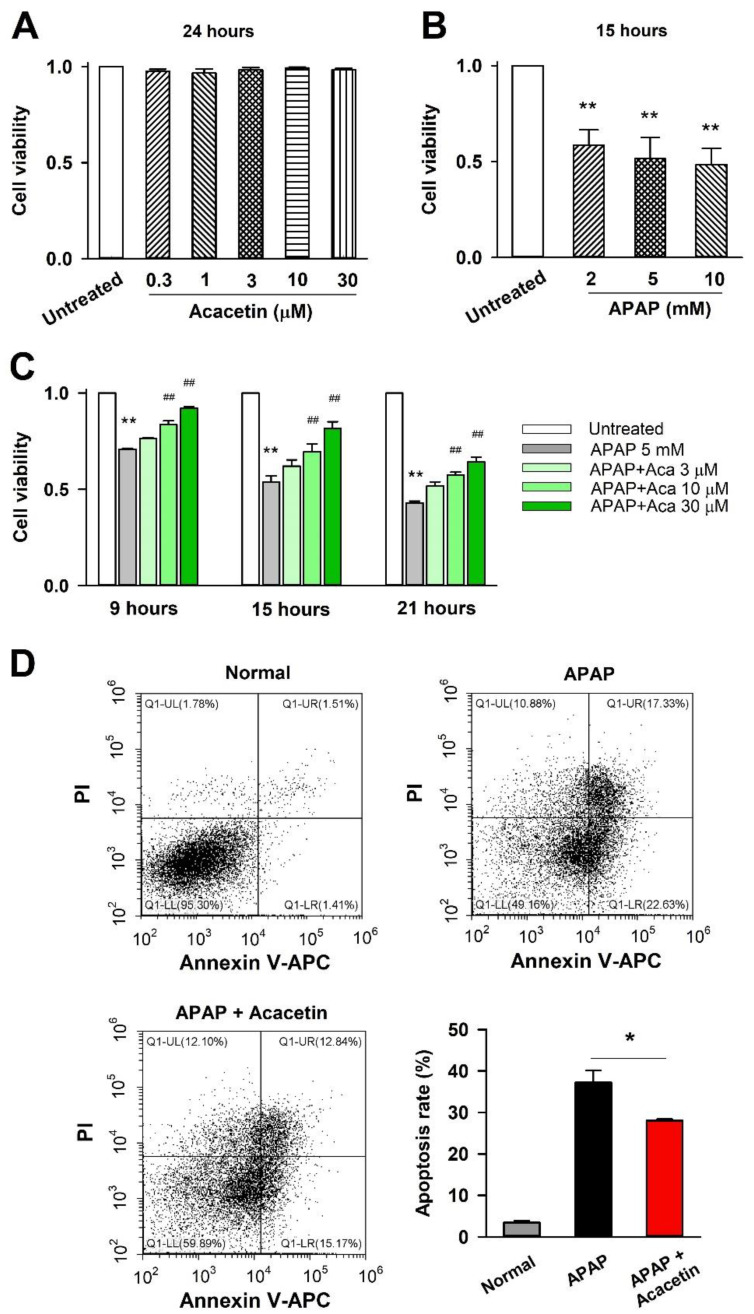
Protective effect of acacetin (Aca) on APAP-treated HepG2 cells. (**A**,**B**) Cell viability evaluated by MTT assay in HepG2 cells treated by various concentrations of acacetin or APAP alone. Cell viability was affected significantly by APAP but not acacetin (** *p* < 0.01 vs. untreated, *n* = 3). (**C**) Cell viability in HepG2 cells incubated with APAP and acacetin in different time periods. Acacetin exhibits the protective effect on APAP-treated HepG2 cells (** *p* < 0.01 vs. untreated, ^##^ *p* < 0.01 vs. APAP, *n* = 3). (**D**) HepG2 cell apoptosis tested by flow cytometry assay. Acacetin inhibits cell apoptosis induced by APAP. Data are expressed as mean ± SEM (* *p* < 0.05 vs. APAP, *n* = 3).

Figure 1 displays the structures of acacetin and acacetin prodrug. The prodrug is the sodium phosphate of acacetin. In vitro, HepG2 cells were commonly used to study the effect of some candidate drugs on APAP-induced hepatotoxicity [27,28]. First, we tested the effect of acacetin on HepG2 cell proliferation. The fact that acacetin prodrug does not have the same biological activity as acacetin in vitro has been reported previously [23], so we didn’t examine the effect of acacetin prodrug on HepG2 cells here. Eight hours after seeding, HepG2 cells were incubated with acacetin for another 24 h. Results from MTT suggest that HepG2 cell proliferation was not significantly affected by acacetin in the concentrations of 0.3, 1, 3, 10, and 30 μM (Figure 2A). Figure 2B shows that the cell proliferation was inhibited by APAP in a dose-dependent manner (** *p* < 0.01 vs. untreated, *n* = 3). Here, 5 mM APAP was selected for further experiments. It was found that acacetin could reverse the suppression of APAP on cell proliferation in a dose-dependent manner, but not a time-dependent manner (Figure 2C, ** *p* < 0.01 vs. untreated, ^##^ *p* < 0.01 vs. APAP, *n* = 3). APAP-induced hepatocellular apoptosis has been reported [29]. Flow cytometry was used to test the protective effect of acacetin on cell apoptosis induced by APAP. As shown in Figure 2D, the dots in the upper-right quadrant and lower-right quadrant of each panel represent apoptotic HepG2 cells. Statistical data show that the apoptotic rate of HepG2 cells in the APAP group is 37.2 ± 2.9%, while in the APAP + acacetin (30 μM) group, the apoptotic rate is 28.0 ± 0.4%, which is significantly decreased compared with the APAP group (Figure 2D, * *p* < 0.05). These data strongly indicate the suppressive effect of acacetin on the HepG2 cell apoptosis induced by APAP.

### 2.2. Acacetin Treatment Protects against APAP-Induced Acute Liver Injury

**Figure 3:**
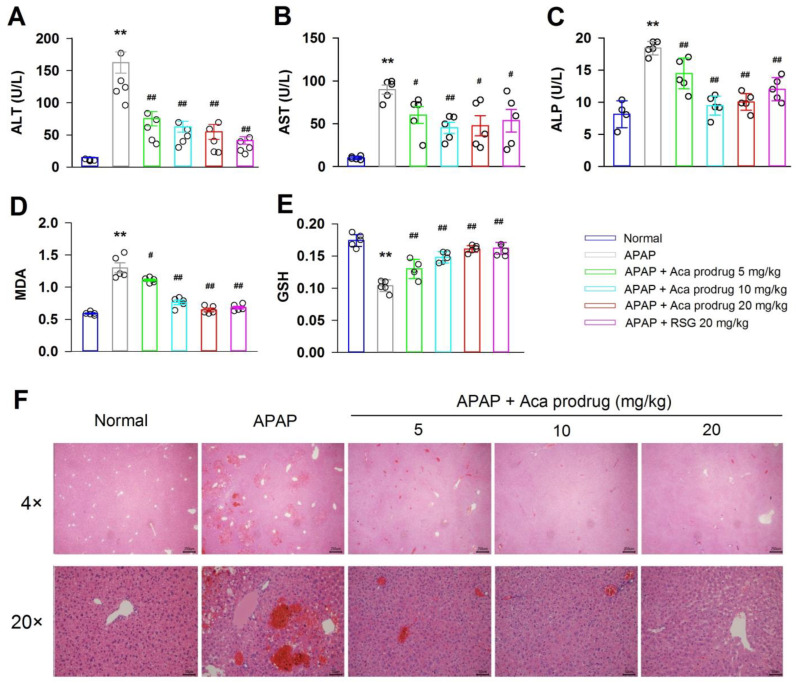
Protective effect of acacetin (Aca) prodrug in vivo. (**A**–**C**) Serum ALT, AST, and ALP activities at 4 h after administration with APAP. (**D**,**E**) MDA activity and GSH levels in mice liver tissue were detected; 5 mice in each group. (**F**) Typical images of HE staining of liver segments (×40 and ×200 magnification). Data are expressed as mean ± SEM (** *p* < 0.01 vs. normal, ^#^ *p* < 0.05, ^##^ *p* < 0.01 vs. APAP, *n* = 5).

The protective effect of acacetin on hepatocytes was evaluated in vivo. Intraperitoneal injection of APAP (300 mg/kg) is common to set up the acute liver injury animal model. After treatment for 4 h, significant increases in serum ALT, AST, and ALP activities and markers of hepatocellular damage were observed in APAP-treated mice compared with normal mice (** *p* < 0.01). However, treatment with acacetin prodrug (5, 10, and 20 mg/kg) by subcutaneous injection reduced the levels of serum ALT, AST, and ALP significantly (^##^ *p* < 0.01). The PPARγ activator rosiglitazone (RSG, 20 mg/kg), which was used as a positive control, also reduced serum ALT, AST, and ALP significantly in APAP-treated mice, as expected (Figure 3A–C).

The effects of acacetin prodrug on hepatic lipid peroxidation during APAP-induced acute liver injury were analyzed. As shown in Figure 3D, the level of hepatic MDA, a marker of lipid peroxidation, was significantly elevated after APAP injection. However, the elevation of MDA was attenuated by acacetin prodrug treatment in a dose-dependent manner. In addition, the effects of acacetin prodrug on APAP-induced hepatic GSH depletion were also evaluated, and the result is shown in Figure 3E. Hepatic GSH content was significantly reduced after APAP injection, but it was restored after treatment with acacetin prodrug (Figure 3D,E, ** *p* < 0.01 vs. normal, ^#^ *p* < 0.05, ^##^ *p* < 0.01 vs. APAP).

Histological analysis of liver samples with hematoxylin and eosin (H&E) staining strongly supported the protective effect of acacetin prodrug on hepatocytes. As shown in Figure 3F, there was no pathological abnormality in the normal group livers. However, liver sections isolated at 4 h after APAP treatment showed multiple areas of portal inflammation and severe hepatocellular necrosis, as well as inflammatory cell infiltration. This histologic damage was significantly alleviated by treatment with acacetin prodrug (5, 10, and 20 mg/kg). Histological examination indicated that acacetin prodrug could effectively protect the liver against APAP-induced hepatocellular damage.

### 2.3. Acacetin Suppresses ER Stress and Apoptosis in APAP-Induced Liver Injury

**Figure 4:**
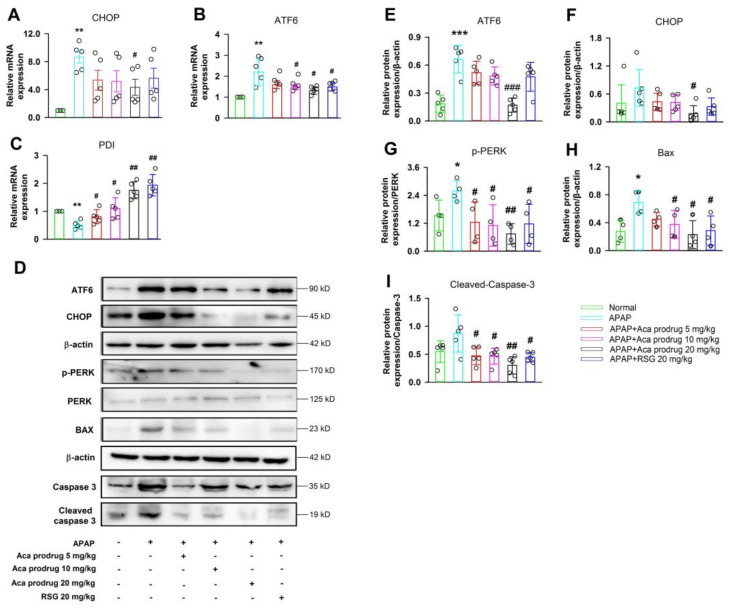
Acacetin (Aca) prodrug alleviates APAP-induced ER stress and apoptosis in liver. (**A–C**) mRNA levels of CHOP, ATF6, and PDI determined by real-time PCR in livers after administration with APAP. (**D**) Representative results of ATF6, CHOP, p-PERK, PERK, BAX, Caspase-3, and cleaved caspase-3 protein expressions were determined by Western blot in livers after administration with APAP. (**E–I**) Statistical analysis of the results of Western blot. The experiments were repeated in 5 samples of each group. Data are expressed as mean ± SEM (* *p* < 0.05, ** *p* < 0.01, *** *p* < 0.001 vs. normal, ^#^ *p* < 0.05, ^##^ *p* < 0.01, ^###^ *p* < 0.01 vs. APAP).

ER stress was reported to play an important role in APAP-induced acute liver injury in mice. To explore whether acacetin prodrug treatment alleviated ER stress caused by APAP administration, the gene and/or protein expressions of CHOP, ATF6, PDI, and p-PERK in livers were measured. In addition, the protein expressions of BAX, Caspase-3, and cleaved caspase-3 were also analyzed. Figure 4 shows these gene and protein expressions in APAP mice were much higher than those in the normal group, except that PDI, which is considered as a suppressor of ER stress and apoptosis, was lower in APAP mice compared with normal mice. However, acacetin prodrug, especially at 10 or 20 mg/kg, could significantly reverse the changes in these gene and protein expressions. Similar results were observed in the RSG group. These data suggest that acacetin prodrug treatment ameliorates APAP-induced liver ER stress and hepatocellular apoptosis (Figure 4A–C,E–I, * *p* < 0.05, ** *p* < 0.01, *** *p* < 0.001 vs. normal, ^#^ *p* < 0.05, ^##^ *p* < 0.01, ^###^ *p* < 0.001 vs. APAP).

### 2.4. Acacetin Upregulates PPARγ and Suppresses ER Stress In Vitro

**Figure 5:**
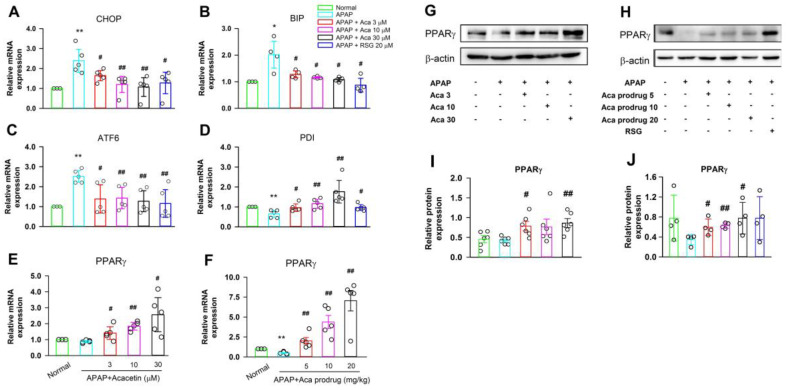
Acacetin (Aca) alleviates APAP-induced ER stress and upregulates PPARγ in HepG2 cells. (**A–D**) mRNA levels of CHOP, BIP, ATF6, and PDI were determined by real-time PCR in HepG2 cells incubated with APAP and acacetin. (**E**,**F**) mRNA level of PPARγ determined by real-time PCR in HepG2 cells and livers after administration with APAP. (**G**,**H**) Representative results of PPARγ protein expression were determined by Western blot in HepG2 cells and livers. The experiments were repeated in 3 samples of each group. (**I**,**J**) Statistical analysis of the results of Western blot. Data are expressed as mean ± SEM (* *p* < 0.05, ** *p* < 0.01 vs. normal, ^#^ *p* < 0.05, ^##^ *p* < 0.01 vs. APAP, *n* = 4–5).

To study the mechanism of how acacetin suppresses ER stress, HepG2 cells were treated with APAP or APAP + acacetin for 15 h. First, the changes in gene expression of CHOP, ATF6, BIP, and PDI were reversed by acacetin in a dose-dependent manner in HepG2 cells treated with APAP (Figure 5A–D, * *p* < 0.05, ** *p* < 0.01 vs. untreated, ^#^ *p* < 0.05, ^##^ *p* < 0.01 vs. APAP). These results are consistent with the observations in mice. Subsequently, we found that PPARγ was downregulated by APAP both in vivo and in vitro. However, this downregulation could be significantly reversed by acacetin in vitro or acacetin prodrug in vivo (Figure 5E–J, ** *p* < 0.01, vs. normal, ^#^ *p* < 0.05, ^##^ *p* < 0.01, vs. APAP).

### 2.5. Suppressive Effect of Acacetin on ER Stress Was Alleviated by Knockdown or Inhibition of PPARγ

**Figure 6:**
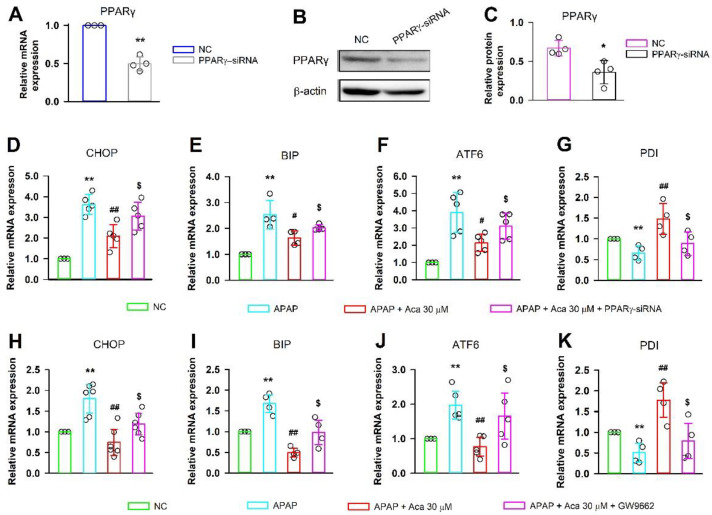
PPARγ-siRNA or PPARγ inhibitor GW9662 reverses the protective effects of acacetin (Aca) against APAP-induced ER stress in HepG2 cells. (**A**,**B**) mRNA and protein level of PPARγ determined by real-time PCR and Western blot in HepG2 cells incubated with specific PPARγ-siRNA. (**C**) Quantification of PPARγ protein in HepG2 cells incubated with specific PPARγ-siRNA. (**D**–**G**) mRNA levels of CHOP, BIP, ATF6, and PDI were determined by real-time PCR in HepG2 cells incubated with PPARγ-siRNA. (**H**–**K**) mRNA levels of CHOP, BIP, ATF6, and PDI were determined by real-time PCR in HepG2 cells incubated with GW9662. Data are expressed as mean ± SEM (* *p* < 0.05, ** *p* < 0.01 vs. NC, ^#^ *p* < 0.05, ^##^ *p* < 0.01 vs. APAP, ^$^ *p* < 0.05 vs. acacetin, *n* = 4–5). NC: negative control.

**Figure 7:**
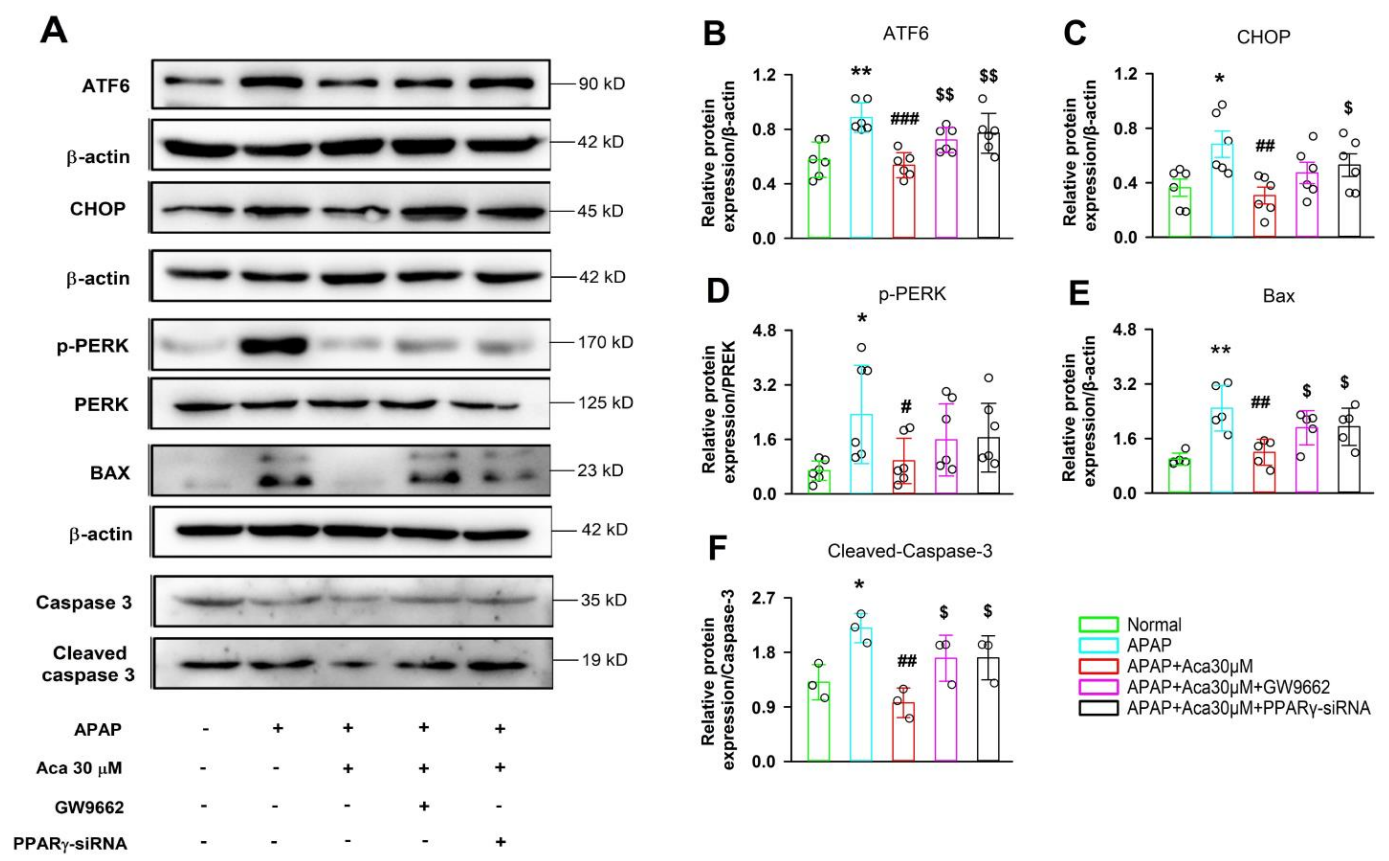
(**A**) Representative results of ATF6, CHOP, p-PERK, PERK, BAX, Caspase-3, and cleaved caspase-3 protein expressions were determined by Western blot in HepG2 cells after administration with PPARγ-siRNA or PPARγ inhibitor GW9662. (**B**–**F**) Statistical analysis of the results of Western blot. The experiments were repeated in 5 samples of each group (* *p* < 0.05, ** *p* < 0.01 vs. normal, ^#^ *p* < 0.05, ^##^ *p* < 0.01, ^###^ *p* < 0.001 vs. APAP, ^$^ *p* < 0.05, ^$$^ *p* < 0.01 vs. acacetin).

Specific small interfering RNA and inhibitor GW9662 were employed to explore whether PPARγ participates in the suppressive effect of acacetin on ER stress induced by APAP. First, the effect of siRNA on PPARγ expression was tested in HepG2 cells, the gene and protein expression of PPARγ were significantly decreased compared with the negative control (Figure 6A–C, * *p* < 0.05, ** *p* < 0.01). As expected, the APAP-induced high gene expressions of CHOP, BIP, and ATF6 were decreased by acacetin (30 μM), but these decreases were abolished by PPARγ-siRNA or GW9662; the expression of PDI, a suppressor of ER stress, was decreased by PPARγ-siRNA or GW9662 compared with acacetin (Figure 6D–K, ** *p* < 0.01 vs. NC, ^#^ *p* < 0.05, ^##^ *p* < 0.01 vs. APAP, ^$^ *p* < 0.05 vs. acacetin). The protein expressions of ATF6, CHOP, p-PERK, PERK, BAX, Caspase-3, and cleaved caspase-3 were measured in cells treated with APAP, APAP + acacetin, APAP + acacetin + GW9662, and APAP + acacetin + PPARγ-siRNA. Figure 7 shows that PPARγ-siRNA or GW9662 can reverse the downregulation of these proteins caused by acacetin (Figure 7B–F, * *p* < 0.05, ** *p* < 0.01 vs. normal, ^#^ *p* < 0.05, ^##^ *p* < 0.01, ^###^ *p* < 0.001 vs. APAP, ^$^ *p* < 0.05, ^$$^ *p* < 0.01 vs. acacetin). In order to further confirm the important role of PPARγ in AILI, GW9662 was employed to treat HepG2 cells incubated with APAP and/or acacetin, and then cell viability was evaluated. It was found that GW9662 could abolish the protective effect of acacetin on HepG2 cells (supplementary Figure S1). These data provide evidence that PPARγ is required for the suppressive effect of acacetin on ER stress and apoptosis induced by APAP.

### 2.6. The Quantum Chemical Calculation and Molecular Dynamics Simulation Analysis

**Figure 8:**
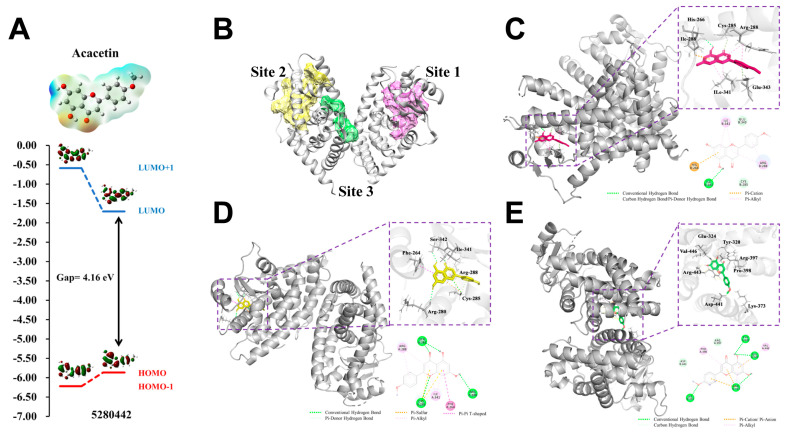
Schematic 2D and 3D representation that the molecular electrostatic potential and electronic properties of acacetin (**A**), the active pockets (**B**), the molecular docking model, active sites and binding distances, and the ray tracing of compound acacetin in the protein PPARγ (PDB ID: 1KUN) at site 1 (**C**), site 2 (**D**), and site 3 (**E**), respectively.

The Molecular Electrostatic Potential (MEP) map of acacetin provides a visual representation of the electronic density, chemically active sites, and the reactivity of the atoms. The different values of electrostatic potential at the MEP surface are represented by different colors: red, blue, and green represent the regions of most negative, most positive and zero electrostatic potential, respectively. Specifically, the MEP of acacetin clearly shows one major negative potential region around the oxygen atoms of the carbonyl group (represented by red color). The H atom indicates the strong attraction and the oxygen atom indicates the repulsion. The value of the electrostatic potential is largely responsible for the binding of a substrate to its receptor binding sites and thus leads to further development of the receptor-ligand interaction (Figure 8A upper).

To further explore the energetic behavior, the 3D plots of the HOMO, HOMO + 1, LUMO and LUMO + 1 frontier orbitals of acacetin are depicted in Figure 8 and the HOMO-LUMO energy calculated results are given in Table 1. As the most important orbitals in molecules, the highest occupied molecular orbital (HOMO) represents the ability to donate an electron, and the lowest unoccupied molecular orbital (LUMO) represents the ability to obtain an electron; these are useful to understand the chemical reactivity and kinetic stability of acacetin. As shown in Figure 8, the positive phase is presented in red and the negative phase is in green. For the HOMO and LUMO molecular orbitals, the charge density is localized over the entire molecules for acacetin. Moreover, the energy levels of HOMO − 1, HOMO, LUMO, and LUMO  +  1, as well as the HOMO-LUMO band gaps, were computed and are shown in Table 1. The HOMO-LUMO gap energies calculated at the B3LYP/6-311++G (d, p) level are 4.1614 eV, which implies high molecular stability in the sense of its lower reactivity in chemical reactions. The chemical reactivity descriptions, chemical potential (μ), chemical hardness (η), and chemical softness (σ) were defined to get a deeper understanding of the ligand-binding phenomena. The chemical hardness (η) of acacetin is 2.0807 and the chemical softness (σ) is 0.4806; the large HOMO-LUMO gap indicates a hard molecule with lower reactivity. The chemical potential (μ) of acacetin is 3.7852, which remains constant throughout the system and reflects the property of the entire molecule.

In order to verify the functional effects of acacetin in non-alcoholic fatty liver, the interaction between acacetin and PPARγ was tested and verified randomly. First, the active pockets were speculated by DoGisteScorer (Figure 8B). Then, the interaction between acacetin and PPARγ were elucidated by AutoDockTools-1.5.6, Discovery Studio 4.5, and Pymol software. Notably, the lower binding energy meant that the bindings between the compounds and the targets were stronger. The hydrogen bonding and aromatic stackings (Pi-sigma, Pi-alkyl, Pi-anion, Pi-sulfur, and Pi-cation interactions) were found to be involved in the active site residues and acacetin. The 3D mode and 2D dimensional representation of acacetin in the three active pockets of PPARγ (1KUN) are displayed in Figure 8C–E. The free binding energy of acacetin with PPARγ at active site 1 was found to be −7.46 kcal/mol. In addition, acacetin binds to Ile281, Cys285, Glu343, His266, Arg288, and Ile341 by conventional hydrogen bond, carbon hydrogen bond, Pi-donor hydrogen bond, one Pi-cation, and one Pi-alkyl, respectively. The interaction types of binding complexes are also depicted in the enlarged drawing in Figure 8C. Similarly, the free binding energy of acacetin with PPARγ at active sites 2 and 3 were found to be −6.64 and −5.57 kcal/mol, respectively. At active pocket 2, acacetin binds to Ser342, Cys285, and Arg280 by conventional hydrogen bond, binds to Phe264 by one Pi-Pi T-shaped bond, and binds to Arg288, Ile341 and Cys285 by other aromatic stacking. At active pocket 3, there are 6 hydrogen bonds between acacetin and Asp441, Arg397, Lys373, Arg443, Glu324, and Try320, and strong aromatic stacking interactions with Pro398, Val446, and Arg443. The interaction types and distance of these binding complexes are also depicted in the ray-tracing diagram in Figure 8C–E.

## 3. Discussion

The present study investigated the therapeutic effect of a water-soluble acacetin prodrug, sodium phosphate of acacetin, on AILI. The data showed that acacetin administration in vitro significantly attenuated APAP-induced HepG2 cell apoptosis. Acacetin prodrug treatment in vivo decreased APAP-induced elevation of serum ALT, AST, and ALP levels. MDA and GSH in liver tissue were also analyzed, and the increase in MDA or hepatic GSH depletion caused by APAP were restored after acacetin prodrug treatment. It has been reported that ER stress is an important event during AILI [11,20]. Our study showed that acacetin/acacetin prodrug alleviated ER stress induced by APAP in a dose-dependent manner both in vivo and in vitro. In addition, PPARγ-siRNA and GW9662 were used to demonstrate that PPARγ was required for acacetin-suppressing ER stress. Moreover, computer molecular docking further uncovered the interaction between acacetin and PPARγ. These results demonstrated the effect of acacetin prodrug on preventing the progression of AILI via activating PPARγ and suppressing ER stress and indicated the potential therapeutic value of acacetin prodrug for AILI in clinic in the future.

Although PPARγ is predominantly expressed in adipocytes, where it plays an essential role in regulation of adipogenesis; it is also expressed in many other cells, including hepatocytes, smooth muscle cells, and macrophages. A number of studies have revealed the regulatory role of PPARγ in apoptosis and inflammation [30,31]. However, the role of PPARγ in APAP-induced acute liver injury is unclear. Only two publications reported the protective effects of PPARγ agonists pioglitazone and rosiglitazone against AILI [32,33]. However, whether PPARγ is involved in the protective effects of pioglitazone or rosiglitazone is still unknown. Of interest, our present study found that PPARγ is downregulated in hepatocytes after APAP treatment, indicating the protective role of PPARγ in AILI. This finding may also help to explain the effects of pioglitazone and rosiglitazone reported previously. Although the concept that PPARγ works as a negative regulator of ER stress was reported in some publications [34,35,36], we revealed the relationship between PPARγ and ER stress for the first time in AILI.

ER stress is an important intrinsic stress of integrated stress response (ISR), which is a conserved intra-cellular signaling network and promotes cell survival and homeostasis. Both intrinsic and extrinsic stresses are sensed by four specialized kinases: PERK, GCN2, PKR, and HRI. Subsequently, the central player of ISR elF2α is phosphorylated [37]. At the same time, ISR could be activated by the changes of cellular amino acid metabolism, causing alterations of pro-survival or pro-apoptotic gene expression. The important role of ISR in the relationship between essential amino acid limitation and health promotion was well reviewed by Dr. Angelos Sikalidis [38]. A recent study revealed the effect of dietary restriction on alleviating AILI via stimulating GSH synthesis [39]. Our results showed that acacetin prodrug treatment significantly reversed the decrease of hepatic GSH activities in a dose-dependent manner during AILI. However, the mechanism is unclear. Whether acacetin prodrug causing the restoration of GSH during APAP treatment is associated with the amino acid metabolism and ISR needs further study.

A previous publication reported the protective effects of acacetin on D-galactosamine and LPS-induced fulminant hepatic failure in mice, in which acacetin was dissolved in 5% DMSO-saline solution and administrated by intraperitoneal injection. The dose of acacetin used in that study was as high as 100 mg/kg, and their results show that acacetin protects against liver injury via suppressing TLR4 signaling and enhancing autophagic flux [25]. In addition, oral administration of apigenin 80–100 mg/kg, which has a similar structure with acacetin, could also prevent D-GalN/LPS or AILI; the mechanism might be associated with Nrf-2-mediated antioxidative enzyme, PPARγ/NF-κB-mediated inflammation, and the SIRT1-p53 axis [26,40]. Our present study demonstrated a water-soluble acacetin prodrug which was administered by subcutaneous injection exhibiting similar protective effects on AILI. More importantly, the effective dosage of acacetin prodrug in our study is as low as 5 mg/kg, indicating that the bioavailability of our prodrug is much higher than that of acacetin administered by intraperitoneal injection. In addition, we provided additional evidence and mechanisms to confirm the protective effects of acacetin prodrug on AILI. The molecular docking shows that acacetin can bind to three active pockets of PPARγ by conventional hydrogen bond, carbon hydrogen bond, Pi-donor hydrogen bond, and Pi-Pi T-shaped bond.

The biggest contradiction of the studies on natural flavone including acacetin is that it is very easy to obtain results from cell culture or enzyme assays; however, they may not have real biological significance because of poor bioavailability and rapid metabolism in animal experiments [41]. Therefore, very few flavones can go to market for clinical application. Modifying the chemical structure of flavones to improve bioavailability may be a good strategy. Hence, in order to improve the solubility and absorption of acacetin, many efforts have been made by our group to modify the structure of acacetin. Finally, we obtained the water-soluble acacetin prodrug, and at the same time, the diverse pharmacological activities of acacetin were reserved. Our results suggest that acacetin prodrug is a promising candidate for clinical application.

Of note, CYP 2E1 is also expressed in the kidneys, so kidney injury is a common event in APAP overdose. In fact, acute kidney injury could contribute to the increased morbidity and mortality observed in AILI [42]. The normal function of kidneys is very important in AILI, but NAC, the only marketed medicine for treatment of AILI, cannot effectively protect against APAP-mediated kidney injury [43]. Previous studies have reported the protective effect of acacetin on kidneys suffering from type 2 diabetes and ischemia reperfusion via antioxidant and anti-inflammation. So, it makes sense to evaluate the protective effect of acacetin prodrug on kidney injury induced by APAP.

However, the present study has several limitations. First, in vivo study is needed to confirm the molecular target of acacetin, for example, PPARγ knockout mice should be employed to evaluate the effect of acacetin. In addition, although computer molecular docking displays the interaction between PPARγ and acacetin, this should be further demonstrated with specific experiments. Second, it is still unclear how ER stress is regulated by PPARγ. We don’t know whether PPARγ regulates ER stress directly or indirectly. In addition, the toxicity of the compound also needs to be evaluated.

In summary, the present study demonstrated the protective effects of acacetin prodrug on AILI. Our results provide evidence that acacetin prodrug targets PPARγ in modulation of ER stress in the progression of AILI. Thus, acacetin prodrug is a promising candidate for treatment of liver injury in future.

## 4. Materials and Methods

### 4.1. Chemical Reagents

Both acacetin and the water-soluble acacetin prodrug were kindly provided by Dr. Gui-Rong Li from Nanjing Amazigh Pharma Ltd. (Nanjing, China). The synthesis process and in vivo conversion of the acacetin prodrug were reported previously [23,24]. APAP, dimethyl sulfoxide (DMSO), MTT, rosiglitazone (RSG), and GW9662 were purchased from Sigma-Aldrich Chemicals (St. Louis, MO, USA). For the cell experiment, acacetin was dissolved with DMSO in a concentration of 30 mM and stored at −20 °C.

### 4.2. Cell Culture

Human liver cancer cell line HepG2 provided by Procell Life Science & Technology Co., Ltd. (Wuhan, China) was cultured in high-glucose DMEM supplemented with 10% fetal bovine serum (FBS), 100 μg/mL penicillin, and 100 μg/mL streptomycin (Invitrogen, Shanghai, China) and maintained in a cell incubator containing 5% CO_2_ at 37 °C. The culture medium was changed every two days with a conventional cell culture procedure.

### 4.3. Cell Viability Assay

HepG2 cells were seeded in 96-well plates at a density of 1.8 × 10^4^ cells per well for 8 h, then cultured in the medium containing APAP, acacetin, or RSG for different time periods. MTT (20 μL, 5 mg/mL) solution was then added to each well and incubated at 37 °C under dark for 4 h. Then the medium was removed and DMSO was applied to each well. The plates were read using a microplate spectrophotometer at 570 nm with 630 nm as a reference. Cell viability was normalized as a percentage of control wells.

### 4.4. Animal Experiments

Six-week-old ICR male mice were purchased from the Shanghai Experimental Animal Center (Shanghai, China). The standard animal diet and water were given according to a 12 h light-dark cycle at the Experimental Animal Center of Ningbo University under the condition of controlled temperature (22 °C). The experimental procedure was approved by the Committee on the Use of Live Animals in Teaching and Research of Ningbo University (Approved No: 2019-48).

After adaptive feeding for 1 week, mice were randomly divided into six groups: vehicle control (normal), APAP (300 mg/kg), APAP (300 mg/kg) + acacetin prodrug (5 mg/kg), APAP (300 mg/kg) + acacetin prodrug (10 mg/kg), APAP (300 mg/kg) + acacetin prodrug (20 mg/kg), and APAP (300 mg/kg) + rosiglitazone (20 mg/kg); there were 5 mice in each group. APAP, acacetin prodrug, and rosiglitazone were dissolved in 0.9% saline. After the mice fasted for 16 h, acacetin prodrug and rosiglitazone were administered via subcutaneous injection and intraperitoneal injection, respectively. After 1 h, APAP was administrated by intraperitoneal injection. Another 4 h later, all mice were sacrificed and blood and livers were collected for blood biochemical and molecular biology analysis.

### 4.5. ALT, AST, ALP, MDA, and GSH Measurement

The blood samples were kept at room temperature for 30 min. After centrifugation at 1000× *g* for 10 min at 4 °C, the supernatant was collected for analysis. Liver tissues were homogenized in PBS buffer and centrifuged at 2500× *g* for 10 min at 4 °C, then the supernatant was collected. The levels of alanine aminotransferase (ALT), aspartate aminotransferase (AST), alkaline phosphatase (ALP), malondialdehyde (MDA), and glutathione peroxidase (GSH) were detected according to the manuals of the kits which were purchased from Nanjing Jiancheng Institute of Bioengineering (Nanjing, China).

### 4.6. Histopathological Analysis

Liver samples were fixed with formalin, dehydrated, embedded in paraffin, sliced, and stained with H&E. The pathological changes were observed under a light microscope and photographed with 200× magnification. Finally, the degree of liver injury and structural changes were evaluated.

### 4.7. Quantitative Real-Time PCR Analysis

Total RNA was isolated using the TRIzol method (Invitrogen, China), and RNA concentration was measured using a NanoDrop 2000 and normalized among the experimental sample set. cDNA was generated using the PrimeScript^TM^ reverse transcript (RT) reagent Kit (Takara, Beijing, China) used for quantitative real-time PCR. Quantitative RT-PCR reactions were prepared with SYBR Premix Ex Taq^TM^ II (Tkara, Beijing, China) and primers specific for human or mouse GAPDH, CHOP, BIP, ATF6, PDI, and PPARγ (Table S1). The reactions were run in a Lightcycler 480 real-time PCR detection system (Roche, Indianapolis, IN, USA) according to the manufacturer’s instructions. Relative mRNA levels were determined using the ΔΔCt method and normalized to GAPDH.

### 4.8. Western Blotting Analysis

The Western blotting was performed with the procedure described previously. Briefly, 20 mg liver tissue or HepG2 cells were lysed with modified RIPA buffer for 30 min on ice; cell lysates were then centrifuged at 12,000× *g* for 20 min at 4 °C. After transferring the supernatant to a fresh ice-cold tube, protein concentration was quantified with a BCA assay kit, then mixed with SDS sample buffer and denatured at 95 °C for 5 min; samples were resolved with 10% SDS-page gel. Gels were then transferred onto PVDF membrane papers and the membranes were blocked with 5% non-fat milk in TTBS for 1 h. Then membranes were incubated with primary antibodies overnight at 4 °C. Anti-β-actin (1:1000, #66009-1-Ig) was purchased from proteintech, Wuhan, China. Anti-CHOP/GADD (1:800, sc-7351), anti-ATF6 (1:800, sc-166659), anti-Caspase-3 (1:200, sc-56053), anti-Bax (1:200, sc-7480), anti-PERK (1:100, sc-377400), and anti-PPARγ (1:600, sc-7273) antibodies were purchased from SantaCruz Biotechnology. Anti-p-PERK (1:100, #3179) was purchased from Cell Signaling Technology. After washing with TTBS, the membranes were incubated with HRP-conjugated secondary antibodies for 1 h. The membranes were washed again with TTBS and then the blots were analyzed using an enhanced chemiluminescence detection system.

### 4.9. Flow Cytometry and Apoptosis Analysis

HepG2 cells were seeded into 33 mm dishes at a density of 1 × 10^6^ cells per dish for 8 h. After incubation in the medium containing APAP, acacetin, and RSG for 15 h, the cells were collected and washed with PBS. Then 1 × 10^5^ cells were resuspended in 100 μL binding buffer containing AnnexinV-APC and PI following the kit manual (Shanghai Bioscience Co., Ltd., Shanghai, China). The cells were detected using a BECKMAN CytoFlex S flow cytometer. For each sample, no less than 10,000 events were acquired.

### 4.10. RNA Interference

Short interference RNA (siRNA) molecules targeting human PPARγ (GenePharma, Shanghai, China) had target-specific nucleotides. The siRNA molecules (150 nM) were transfected into HepG2 cells with 60–80% confluence using lipofectamine 2000 (Invitrogen) following the manual. The transfected cells were used for RNA extraction and Western blotting analysis.

### 4.11. Quantum Chemical Calculation of Acacetin

For investigating the quantum chemical properties of acacetin (Pubchem ID: 5280442), the Gaussian 09 program was used to prepare the initial structures and to perform the ab initio calculation at the Becke-3-Lee–Yang–Parr/6-31G level. The geometric optimization of valacyclovir was carried out with the 6-311G++ basis set and B3LYP functional, which were chosen for the high-quality theoretical approach. The frontier molecular orbital parameters, such as the highest occupied molecular orbital (HOMO), the lowest unoccupied molecular orbital (LUMO), HOMO-LUMO energy gaps, the chemical potential (χ), chemical hardness (η), and chemical softness (σ) were calculated based on the outputs of Gaussian 09, in which χ is the value from (E_LUMO_ + E_HOMO_)/2, η is the value from (E_LUMO_ − E_HOMO_)/2, and σ is the value from 1/η. The analysis of frontier molecular orbitals (FMO) and electrostatic potential were also finished by Gauss View.

Electronegativity, chemical potential, chemical hardness, softness, and global electrophilicity index can be calculated by the following equations:

Electronegativity (χ) = (I + A)/2

chemical potential (μ) = −χ

chemical hardness (η) = (I − A)/2

Chemical softness (σ) =1/η

global electrophilicity index (w) = μ^2^/2η

ΔE = E_LUMO_ − E_HOMO_

where I and A are ionization potential and electron affinity, respectively. According to the energies of HOMO and LUMO molecular orbitals, I = −E_HOMO_ and A = −E_LUMO_.

### 4.12. Molecular Docking

To obtain a deeper understanding about the association of active ingredients with PPARγ (Homo sapiens, PDB ID: 1KUN), molecular docking was applied to evaluate the strength and mode of interactions. The crystal structure of PPARγ were obtained from the RCSB Protein Data Bank (PDB, http://www.rcsb.org/ (accessed on 6 June 2022)). The active pockets were identified based on DoGisteScorer (https://proteins.plus/ (accessed on 6 June 2022)). The AutoDock Tools (ADT) graphical user interface was used to prepare the protein by removing water and adding polar hydrogens and the required charges. Autodock 4.2 (http://mgltools.scripps.edu/downloads (accessed on 6 June 2022)) was used to find the minimum binding energy. PyMOL 2.4.0 and Discovery Studio software were performed the dehydration of the receptor protein.

### 4.13. Statistical Analysis

All graphical data were expressed as means ± SEM. Unpaired Student’s t-tests were used as appropriate to evaluate the differences between two group means, and analysis of variance was use for multiple groups. Values of *p* < 0.05 were considered to indicate statistical significance.

## Figures and Tables

**Table 1:** The calculated electronic properties of acacetin.

LUMO + 1 (eV)	LUMO (eV)	HOMO (eV)	HOMO − 1 (eV)
−0.5853	−1.7045	−5.8660	−6.2167
Energy Gap (eV)	Chemical potential (μ)	Chemical hardness (η)	Chemical softness (σ)
4.1614	3.7852	2.0807	0.4806

## Data Availability

The data presented in this study are available on request from the corresponding author. The data are not publicly available due to privacy.

## Supplementary Materials

The following supporting information can be downloaded at: https://www.mdpi.com/article/10.3390/ijms241411320/s1.
