# Supplementary material for: Protective Effect of Water-Soluble Acacetin Prodrug on APAP-Induced Acute Liver Injury Is Associated with Upregulation of PPARγ and Alleviation of ER Stress
Source: Int J Mol Sci. 2023 Jul 11;24(14):11320. doi: 10.3390/ijms241411320 (PMC10380069; doi:10.3390/ijms241411320)
Supplement: Supplementary file 1 [file ijms-24-11320-s001.zip › ijms-2407550-supplementary.pdf]

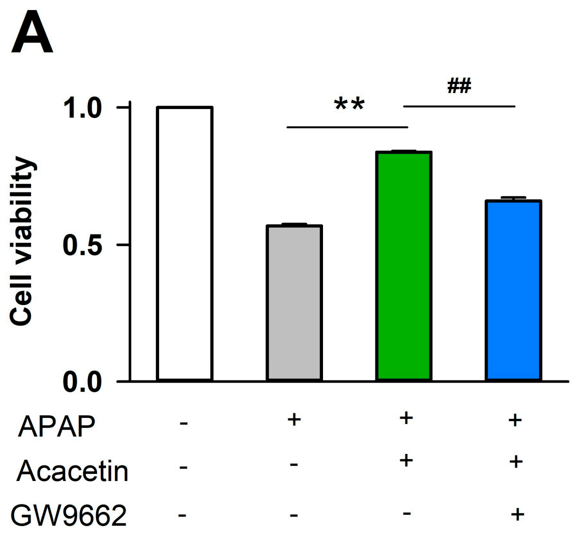

Figure S1. Protective effect of acacetin (Aca) on APAP treated HepG2 cells is reversed by GW9662. (**A**). Cell viability evaluated by MTT assay in HepG2 cells treated by APAP, APAP + acacetin, APAP + acacetin + GW9662 (** $p < 0.01$ vs APAP, ## $p < 0.01$ vs APAP + acacetin, n = 3).

Table S1. primers used for q-PCR analysis.

mGAPDH	F: AGAGTGTTTCCTCGTCCCGT
	R: ACTGTGCCGTTGAATTTGCC
mCHOP	F: GTCACACGCACATCCCAAAG
	R: CACTTTCCGCTCGTTCTCCT
mATF6	F: AGTCGCCTTTTAGTCCGGTTC
	R: ACTCCCAAGGCATCAAATCCA
mPDI	F: GGGGATCACTATATTCCGTCCA
	R: CGGTGAGTAAGTCCTTGCCT
mPPARγ	F: GATCCGTAGAAGCCGTGCAA
	R: TCCTTGGCCCTCTGAGATGA
hGAPDH	F: GTCAAGGCTGAGAACGGGAA
	R: AAATGAGCCCCAGCCTTCTC
hBIP	F: AAGCCCGTCCAGAAAGTGTT
	R: ATCTGGGTTTATGCCACGGG
hCHOP	F: TTCTCTGGCTTGGCTGACTG
	R: TCCTCCTCTTCCTCCTGAGC
hATF6	F: GTATCAGCAGGAACTCAGGGAG
	R: AATGTGTCTCCCCTTCTGCG
hPDI	F: ACCAGCTCGACAAAGATGGG
	R: GAACTCGATGACAAGGGGCA
hPPARγ	F: GCCGAGAAGGAGAAGCTGTT
	R: CTCGCCTTTGCTTTGGTCAG
